# Comparison of the performances of four hydrophilic polymers as supports for lipase immobilisation

**DOI:** 10.1080/13102818.2014.901684

**Published:** 2014-04-30

**Authors:** Lydia Toscano, Gisela Montero, Margarita Stoytcheva, Lourdes Cervantes, Velizar Gochev

**Affiliations:** ^a^ Institute of Engineering, Autonomous University of Baja California, Mexicali, Mexico; ^b^ Technological Institute of Mexicali, Mexicali, Mexico; ^c^ Institute of Agricultural Science, Autonomous University of Baja California, Mexicali, Mexico; ^d^ Department of Biochemistry and Microbiology, Plovdiv University, Plovdiv, Bulgaria

**Keywords:** lipase, adsorption, encapsulation, chitosan, alginate, alginate/PVA copolymer, alginate–chitosan assembly

## Abstract

Four hydrophilic polymers in the form of beads – chitosan, alginate, alginate/polyvinyl alcohol (PVA), and chitosan-coated alginate – were used as supports for lipase immobilisation. Hydrogel beads were characterised by bead-size-distribution estimation, surface morphology studies, and polymer interactions assessment. Matrix performances – loading efficiency, immobilisation yield, enzyme activity, and stability retention – were evaluated and compared. Although the loading efficiency of the chitosan-coated Ca-alginate beads (79.8%) was inferior to that of the Ca-alginate (87%) and of the Ca-alginate/PVA beads (81.3%), their enzyme immobilisation yield (63.96%) was the most important. Moreover, lipase encapsulated in chitosan-coated Ca-alginate beads demonstrated better pH, thermal, and storage (89% residual activity after 30 days) stabilities. Immobilised lipase activity also increased in the order: alginate/PVA > chitosan > alginate > alginate/chitosan, and displayed a maximum at pH 8 and at temperatures of 45 °C (chitosan and Ca-alginate/PVA beads) and 50 °C (Ca-alginate and chitosan-coated Ca-alginate beads). Thus, chitosan-coated Ca-alginate beads could be considered as a suitable support for lipase immobilisation.

## Introduction

Lipases (EC 3.1.1.3 triacylglycerol acylhydrolase), due to their versatility as biocatalysts, able to act at the aqueous and organic interface, and to alter the rate of a number of processes of biotechnological interest, such as triglyceride hydrolysis, esterification, interesterification, transesterification, alcoholysis, acidolysis, and aminolysis, have found a large application in food, detergent, pharmaceutical, leather, textile, cosmetic, paper, and biofuel production industries.[1–3] Nowadays, as an example, biodiesel production by lipase-catalysed transesterification is considered as a promising alternative energy strategy.[4]

Enzymatic catalysis provides several advantages over the chemical one, such as efficiency due to the enzyme specificity and selectivity, reduced energy consumption because of the mild reaction conditions, and limited release of wastes. Additional benefits offer the enzyme immobilisation, resulting from the repeated use of the biocatalyst, its enhanced stability, the localisation of the interaction, and the effective control of the reaction parameters.[5]

The use of immobilised lipases in biodiesel production, the techniques applied for enzyme immobilisation, and the factors affecting the process are extensively reviewed by Stoytcheva et al.[6] Lipase immobilisation is currently performed by adsorption, entrapment and/or encapsulation, and covalent attachment. The entrapment and/or encapsulation are considered as very attractive techniques, because of the common equipment and inexpensive reagents that are used, and the simple immobilisation protocol, preserving the enzyme activity and stability. Various materials have found an application as supports for lipase immobilisation: resins, silica, zeolites, magnetic nanostructures, etc.[6] and a number of alginate and chitosane-based hydrophilic polymers.[7–13] Over the last decades, a variety of such polysaccharide hydrogels have been produced and applied as drug delivery systems,[12,14–17] as carriers for model proteins as hemoglobin,[18] and for controlling the lipid digestion,[19] among others.

The objective of this work is to compare the performances of four hydrophilic polymers in the form of hydrogel beads – chitosan, Ca-alginate, Ca-alginate/PVA, and chitosan-coated Ca-alginate – as supports for lipase immobilisation for their possible application in biodiesel production. Enzyme immobilisation was performed by adsorption and by encapsulation. Polymer beads were characterised, and polymer performances, in terms of loading efficiency and immobilisation yield, enzyme activity, and stability retention, were evaluated.

## Materials and methods

### Materials

All of the used reagents – sodium alginate, chitosan (low molecular weight), polyvinyl alcohol, sodium hydroxide, calcium chloride, boric acid, acetic acid, and so on – were of analytical reagent grade.

Lipase (EC 3.1.1.3) was produced in solid-state fermentation by *Trichoderma harzianum*, as described in a previous work.[20]

### Lipase immobilisation

#### Immobilisation in chitosan beads

Chitosan solution (4% (w/v)) was prepared by dissolving the appropriate amount of chitosan powder in 1% acetic acid. Chitosan beads of various shapes and sizes (3 × 5 mm average) were obtained applying the “syringe method”, i.e. the viscous chitosan solution was extruded (needle diameter 1 mm) dropwise in a coagulant bath containing NaOH (1 mol L^−1^) and ethanol (26% (v/v)) under gentle stirring. The mixture was allowed to remain overnight. Then, the beads were removed by decantation, washed with deionised water until reaching pH 8.0, and soaked in hexane for 1 h under agitation (120 rpm). After hexane excess removal, lipase immobilisation was performed by physical absorption, according to the methodology reported in the literature [8,21] by chitosan beads immersing into a lipase solution for 3 h under stirring at ambient temperature, followed by 24-h static adsorption at 4 °C. The enzyme solution was prepared by dissolving 10 mg of lyophilised lipase into 40 mL of double-distilled water. The obtained derivatives were removed by decantation and filtration, and rinsed with hexane. The decanted solutions and filtrates were collected for estimation of the efficiency of lipase immobilisation. A similar method was used to prepare chitosan beads placebo (without lipase).

#### Immobilisation in Ca-alginate beads

Lipase encapsulation in alginate beads was carried out using established protocols.[13] First, sodium alginate (1.5% (w/v)) solution and CaCl_2_ (0.1 mol L^−1^) were prepared by dissolving suitable amounts of the corresponding reagent in Tris-HCl buffer (0.05 mol L^−1^), pH 8.0. After that, 11.2 mg of lyophilised lipase dissolved in 2 mL of the same buffer was added to 8 mL of the alginate solution and the mixture was gently stirred for 30 min. The obtained lipase containing homogeneous alginate gel was extruded dropwise through a syringe (needle diameter 1 mm) to the gelling solution of CaCl_2_ under stirring. The beads were allowed to polymerise for 40 min. The derivatives were washed twice with 3 mL aliquots of Tris-HCl buffer and the beads were stored at 4 °C for later studies. A similar approach was followed for the preparation of alginate beads placebo. The residual solutions were collected to determine the efficiency of the immobilisation.

#### Immobilisation in chitosan-coated Ca-alginate beads

Lipase encapsulation in alginate was accomplished as described above. Nevertheless, the lipase containing alginate gel was extruded dropwise (needle diameter 1 mm) into a gelling bath obtained by mixing 35 mL of CaCl_2_ aqueous solution (1.5% (w/v)) and 65 mL of chitosan solution (2% (w/v)) in acetic acid. This procedure led to the lipase immobilisation in chitosan-coated alginate beads.[22] The beads were allowed to harden in the bath for 45 min under stirring. The formed derivatives were washed with double-distilled water and stored wet at 4 °C. The residual solutions were analysed for protein content in order to evaluate the enzyme immobilisation efficiency.

#### Immobilisation in hybrid Ca-alginate/PVA beads

The immobilisation was achieved according to the PVA-alginate-boric acid method.[9,10] A homogeneous lipase–copolymer gel was obtained by mixing 10 mL of the alginate solution (2% (w/v)) with 20 mL of the PVA solution (12% (w/v)), and 2 mL of the buffered lipase solution (5.6 mg mL^−1^) under stirring for 30 min. The mixture was extruded (needle diameter 1 mm) as drops into a saturated boric acid solution containing CaCl_2_ (2% (w/v)). The beads were stirred for 45 min to complete the solidification, rinsed with double-distilled water, and finally stored wet at 4 °C. The same procedure was used for placebo preparation. The residual solutions were collected for further analysis.

### Polymer bead characterisation

Bead characterisation implicated: counting of the number of beads obtained in each batch, bead-size-distribution estimation, surface morphology studies, and polymer interaction assessment. The studies were performed using an optical microscope VistaVision and a Fourier transform infrared spectroscopy (FTIR) spectrophotometer WQF FT-IR 520-A.

### Enzyme immobilisation performances of the selected hydrophilic polymers

The polymer loading efficiency and the enzyme immobilisation yield were the two parameters selected to evaluate the enzyme immobilisation performances of the used hydrophilic polymers.

The loading efficiency was defined as the per cent of the immobilised enzyme, and was calculated using the following expression:(1) 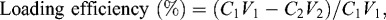
where *C*_1_ and *C*_2_ are the initial and the residual protein concentrations, respectively, and *V*_1_ and *V*_2_ are the initial volume of the enzyme solution and the total volume of the residual solutions, respectively. The initial protein concentration *C*_1_ of the enzyme solutions and the concentration *C*_2_ of the protein remaining in the decanted and filtered solutions and washings were determined according to the Bradford method.[23] Bovine serum albumin was used as a standard.

The immobilisation yield, i.e. the specific activity ratio of immobilised lipase to free lipase, was calculated as follows:(2) 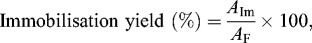
where *A*_Im_ is the specific activity of the immobilised enzyme and *A*_F_ is the specific activity of the free enzyme.

### Enzyme activity and stability assessment

The activity of the free and the equivalent amounts of immobilised lipase was determined spectrophotometrically,[13] using *p*-nitrophenyl butyrate (*p*-NPB) as a lipase substrate. The reaction mixture consisted of 3.9 mL of 0.05 mol L^−1^ Tris-HCl buffer and 0.1 mL of 0.05 mol L^−1^ *p*-NPB. The hydrolysis was achieved for 25 min under stirring (120 rpm). Samples with a volume of 100 μL were withdrawn every 5 min, diluted up to 4 mL, and the released *p*-nitrophenol by the reaction was monitored at 410 nm. Control tests were carried out using the placebo preparations. The enzyme activity was calculated by the initial rate of the enzyme reaction (the slope of the absorbance plot) and the *p*-nitrophenol molar absorption coefficient. The lipase unit was defined as the amount of enzyme that liberates 1 μmol of *p*-nitrophenol per minute under the assay conditions. Enzyme activity was assessed under various experimental conditions, namely pH and temperature.

The thermal and pH enzyme stabilities were estimated by measuring the enzyme activity after thermal treatment, and pH change. The thermal treatment involved sample buffered solution (0.1 mol L^−1^ Tris-HCl, pH 8.0) incubated at 60 °C for 2 h followed by rapid cooling in ice water to reach 25 °C. The pH stability was evaluated by measuring the enzyme activity at pH 8 and 25 °C after sample incubation for 1 h in acetate or 50 mmol L^−1^ Tris-HCl buffers, pH 5.0, 6.0, and 7.0 for 1 h at the same temperature.

The storage stability was determined by residual activity measurements every 10 days (40 °C, pH 8) during a month. Between the measurements, the samples were stored wet at 4 °C. The residual activity was expressed as a per cent of the initial activity.

## Results and discussion

### Polymer bead characterisation

**Figure 1.**
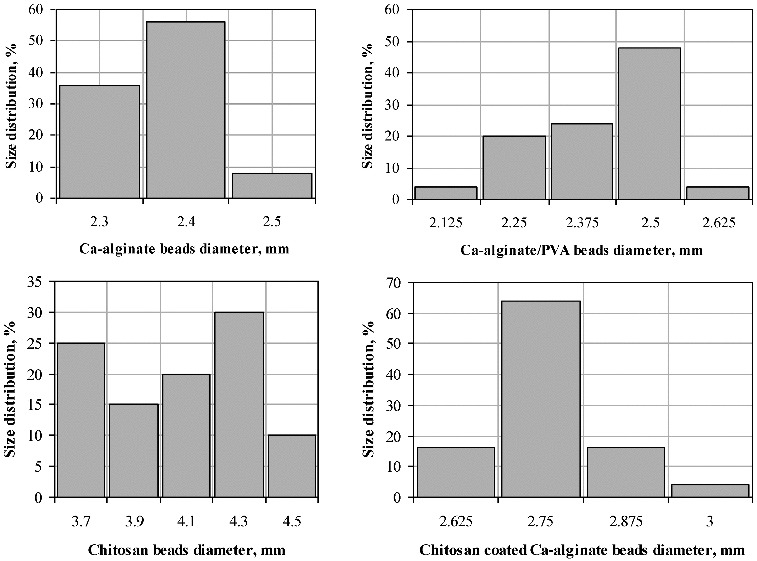
Size distribution of the beads obtained from various hydrophilic polymers.

The number of the obtained polymer beads by applying various methods was approximately 250 per batch. Out of each batch, 25 beads were used for bead-size-distribution estimation. The diameter of each bead was measured in different angles employing an optical microscope (magnification 4x), and the average bead size was calculated. The obtained results are presented in Figure 1. As shown, the size of the Ca-alginate, and of the Ca-alginate/PVA beads, does not differ considerably, varying in the range from 2.125 to 2.625 mm. A total of 56% from the Ca-alginate beads have an average diameter of 2.4 mm, and 48% of the Ca-alginate/PVA beads display an average diameter of 2.5 mm. The diameter of the chitosan-coated Ca-alginate beads is increased to some extent (2.625–3 mm), 64% of the beads having an average diameter of 2.75 mm. As reported in the literature, chitosan binding is affected by the size of the Ca-alginate beads: smaller beads bound more chitosan, due to their higher surface-to-volume ratio.[11] On the other hand, the formation of the alginate–chitosan polyelectrolyte complex involves the carboxyl groups of alginate and the amino groups of chitosan. As demonstrated by Douglas and Tabrizian,[24] the availability of the functional groups in stoichiometric proportion leads to the formation of smaller particles. Nevertheless, the increased relative amount of chitosan provokes an increase in bead size. The pure chitosan beads demonstrate noticeably greater sizes (3.7–4.5 mm) with an average diameter of 3.7 mm for 25% of the beads and an average diameter of 4.3 for 30% of the beads. The average of all the measurements for each of the produced polymers is 2.35, 2.45, 2.75, and 4.35 mm for the Ca-alginate, the Ca-alginate/PVA, the chitosan-coated Ca-alginate, and the chitosan beads, respectively.

**Figure 2.**
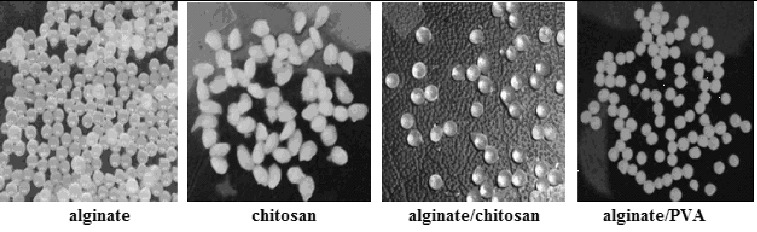
Photos of the hydrophilic polymer beads.

The surface morphology of the beads was studied using optical microscopy (magnification 10x). The observations demonstrated that the Ca-alginate beads, the hybrid Ca-alginate/PVA beads, and the chitosan-coated Ca-alginate beads have a spherical shape, slightly rough surface, compact structure, and elastic properties. In contrast, the obtained chitosan beads were irregularly shaped, with ovoid form and soft deformable texture. The corresponding photos are presented in Figure 2.

**Figure 3.**
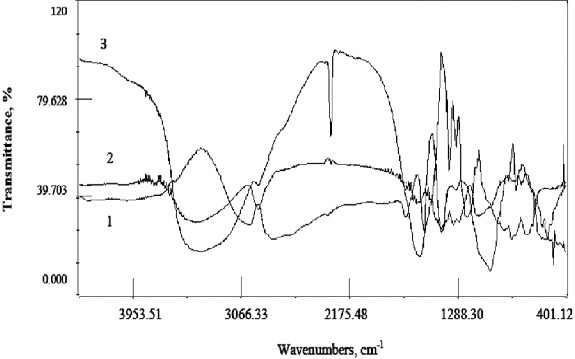
IR spectra: (1) PVA; (2) Ca-alginate/PVA; (3) Ca-alginate.

**Figure 4.**
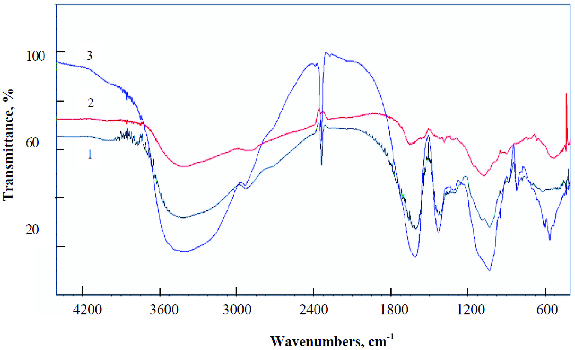
IR spectra: (1) chitosan-coated Ca-alginate; (2) chitosan; (3) Ca-alginate.

The possible interactions between the polymers forming the hybrid alginate/PVA beads and the chitosan-coated alginate beads were investigated by FTIR spectrometry. The recorded spectra are shown in Figures 3 and 4. The bands displayed in the Na-alginate infrared (IR) spectrum at around 1050 and 1640 cm^−1^ are characteristics for the C–O–C stretching attributed to the saccharide alginate structure, and for the asymmetrical stretching of the –COO– groups. The chitosan spectrum shows the characteristic absorption bands of C = O and NH groups at 1633 and 1028 cm^−1^, respectively. The infrared (IR) spectrum of PVA evidences the adsorption bands at 1086 and 1423 cm^−1^, assigned to the C–O stretch. The FTIR spectra of the hybrid alginate/PVA beads and the chitosan-coated alginate beads demonstrate similar spectral features to those of the individual polymers. Therefore, FTIR analysis confirms the absence of strong chemical interactions between the polymers, leading to functional group modification.

### Enzyme immobilisation performances of the selected hydrophilic polymers

As mentioned above, the enzyme immobilisation performances of the selected polymers were quantitatively evaluated by calculating their loading efficiency and immobilisation yield. These two parameters were defined as described in Equations (1) and (2). The data presented in Table 1 demonstrate that the loading efficiency of the Ca-alginate beads is the most significant, and 30% greater than the loading efficiency of the chitosan beads. The lower protein retention of the chitosan matrix could be attributed to various factors such as the immobilisation technique and the polymer structure. Lipase immobilisation in chitosan beads was performed by physical adsorption. As known, the method is simple, but the weak bonds formed favour the enzyme desorption. This effect is combined with the lower diffusion hindrances of the chitosan matrix due to its more open network compared to that of the alginate.[25] It could be expected that the loading efficiency of the Ca-alginate/PVA and of the chitosan-coated Ca-alginate polymer matrices will be increased, because of the improved gel structure of the copolymer,[9] and the reduced permeability of the coated beads.[12] Nevertheless, it was found that their loading efficiencies do not differ noticeably, and are slightly inferior to that of the pure alginate. In contrast, the reached immobilisation yield using the chitosan-coated alginate beads (63.96%) was markedly higher compared to that of the other tested polymers. Obviously, the polymer coating of the alginate beads prevents enzyme from leaking out, and therefore, retains its activity, as reported by Won et al.[13] The poor immobilisation yield of the pure chitosan matrix (23.73%) may be attributed, among other factors, to the larger chitosan bead size. The specific activity of the immobilised enzymes, and hence the immobilisation yield, decreases with increasing bead size due to mass transfer resistance, as reported in the literature.[13]

**Table 1.** Loading efficiency and immobilisation yield evaluation.

Polymer beads	Loading efficiency (%)	Immobilisation yield (%)
Ca-alginate	87.0	44.16
Chitosan	60.7	23.73
Chitosan-coated Ca-alginate	79.8	63.96
Ca-alginate/PVA	81.3	46.57

### Enzyme activity and stability assessment

**Figure 5.**
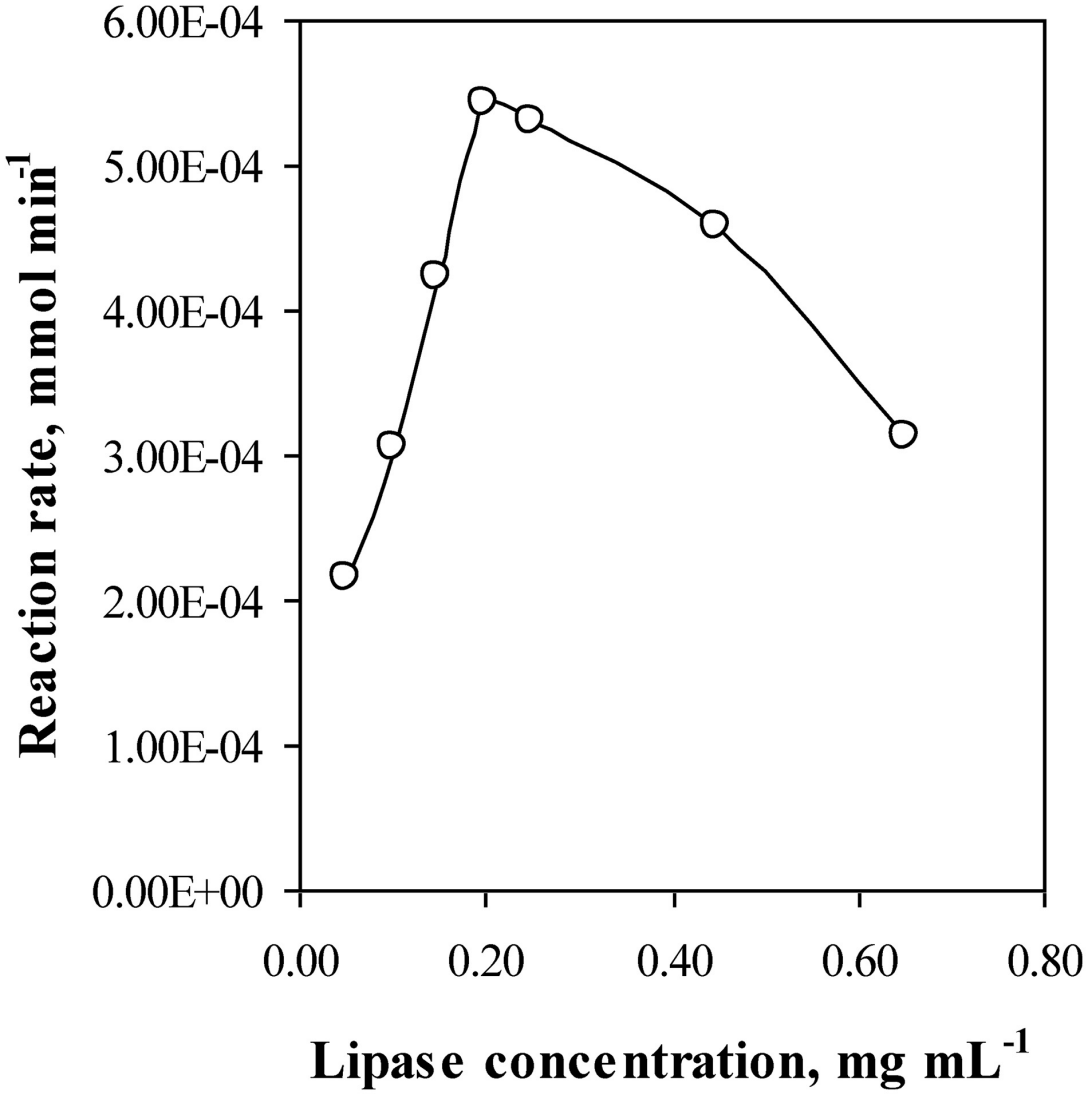
Enzyme concentration dependence of the activity of the free lipase.

Lipase activity evaluation was performed measuring the initial rate of the enzyme-catalysed *p*-NPB hydrolysis. It is noteworthy that the activity of the free lipase increased with enzyme concentration in the studied concentration range (0.05–0.65 mg mL^−1^) only up to 0.20 mg mL^−1^. Then, the lipase activity decreased (Figure 5). A similar behaviour was observed by Fernández et al.[26] and Cruz-Ortiz et al.[9] Activity decrease was attributed to enzyme molecule aggregation, which hinders the catalytic process.

**Figure 6.**
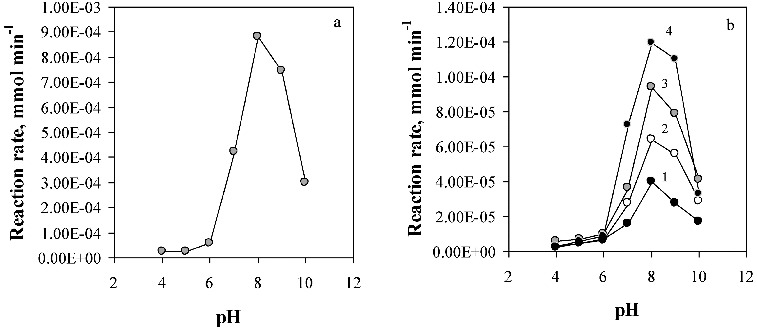
Effect of pH on the activity of the free (a), and immobilised lipase (b). Enzyme supports: (1) Ca-alginate/PVA; (2) chitosan; (3) Ca-alginate; (4) chitosan-coated Ca-alginate beads.

The results, graphically presented in Figure 6, demonstrate the pH effect on the activity of the immobilised lipase using various hydrophilic polymers as enzyme supports compared to that of the free enzyme. The measurements were performed at a temperature of 40 °C in the pH range 4–10, and typical bell-shaped curves were obtained with a maximum at pH 8. Normally, lipases present a maximum activity at neutral or slightly basic solutions.[27]

It was found that the applied method of immobilisation and the nature of the selected support do not affect the pH optimum. Such results were expected, taking into consideration the mild immobilisation conditions and the specific characteristics of the immobilisation applied procedures. Nevertheless, at pH 10 as an example, the free lipase retained only 34% of its optimal activity measured at pH 8, while the retention of the activity of the immobilised lipase was higher, and varied from 42% to 46% of its optimal activity.

**Figure 7.**
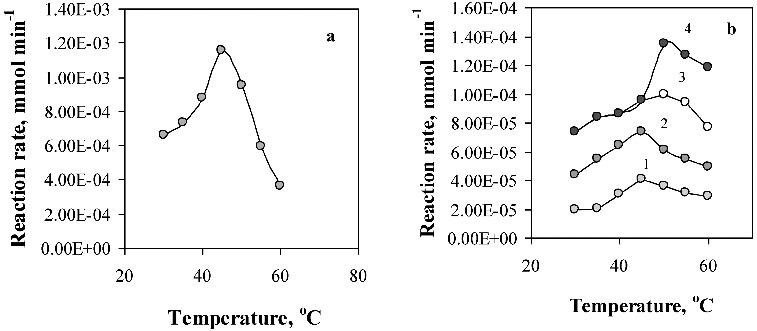
Effect of the temperature on the activity of the free (a), and immobilised lipase (b). Enzyme supports: (1) Ca-alginate/PVA; (2) chitosan; (3) Ca-alginate; (4) chitosan-coated Ca-alginate beads.

The effect of the temperature on the activity of the immobilised lipase was investigated in the temperature range 30–60 °C, and at pH 8 corresponding to the pH optimum (Figure 7). Lipase encapsulated in chitosan and in hybrid Ca-alginate/PVA beads exhibited maximal activity at a temperature of 45 °C, like the free enzyme. At a temperature of 60 °C, a deformation of the hybrid Ca-alginate/PVA beads was observed, followed by their dissolution in the buffer solution. Lipase immobilisation in Ca-alginate and in chitosan-coated Ca-alginate beads resulted in the increase of the lipase activity to some extend with temperature reaching a maximum at 50 °C.

**Figure 8.**
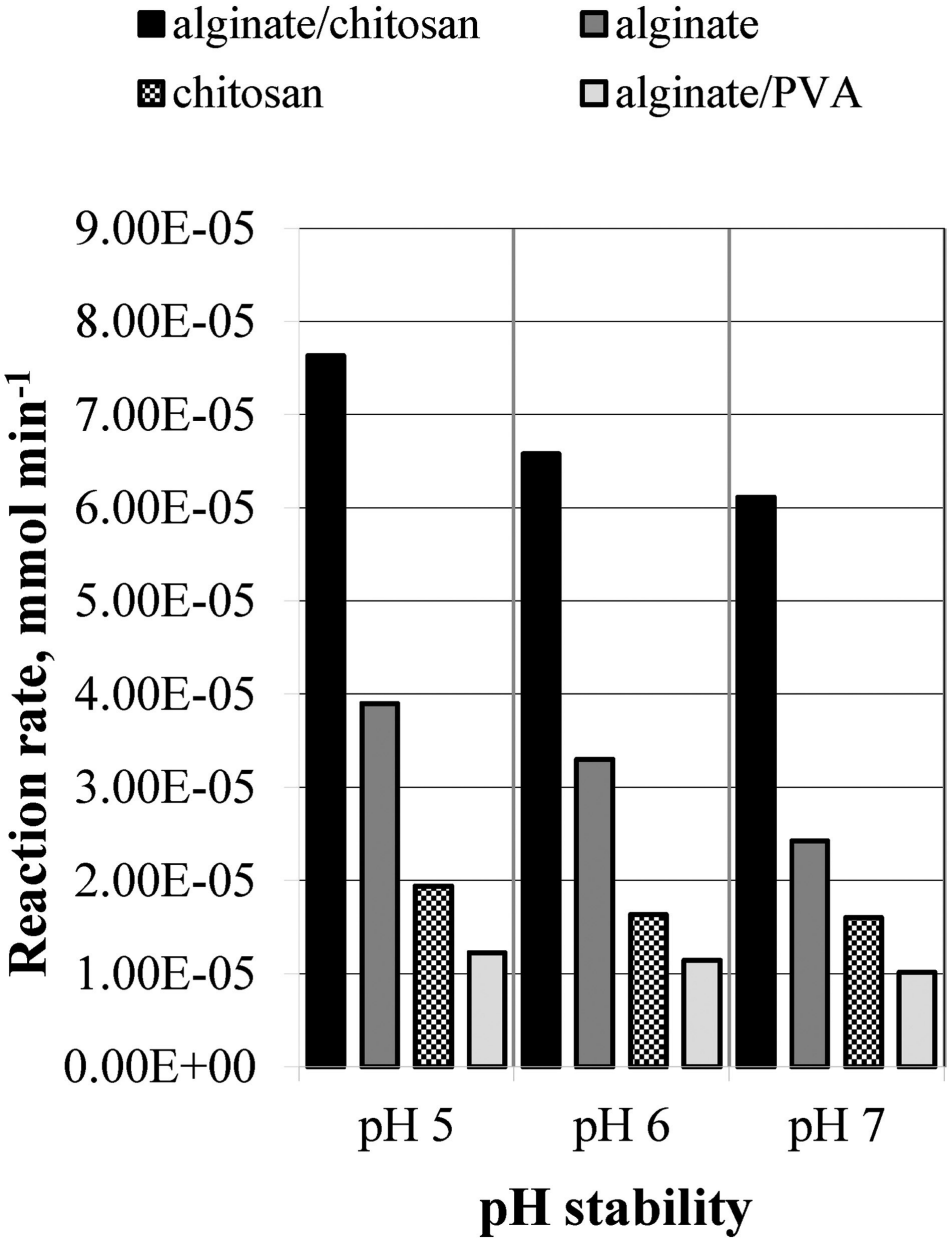
pH stability of the immobilised lipase.

**Figure 9.**
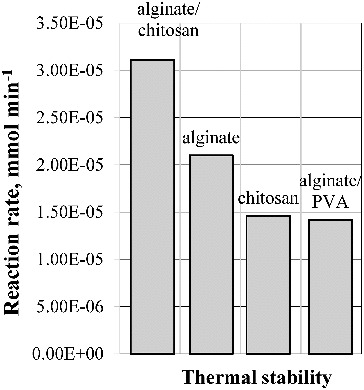
Thermal stability of the immobilised lipase.

All the carried out experiments demonstrated that enzyme activity retention also depends on the nature of the applied support and increases in the range: alginate/PVA > chitosan > alginate > alginate/chitosan. Similar results were obtained estimating the pH and the thermal stability of the immobilised lipase (Figures 8 and 9).

Lipase encapsulated in chitosan-coated Ca-alginate beads conserved 42.08% of its activity after thermal treatment and 99.59% of its activity after incubation at pH 5. Since the process of encapsulation is mostly based on the electrostatic interaction between the negatively charged alginate acid groups and the positively charged chitosan amino groups, it results in the formation of a reinforced gel. Obviously, the restriction of the contact of the enzyme with the environment due to the gel reinforcement favours the retention of its stability and activity.

**Figure 10.**
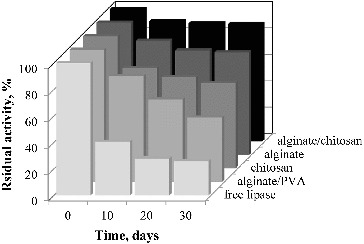
Storage stability of the free and immobilised lipase using various supports.

The evaluation of the storage stability of the immobilised lipase demonstrated that it increases in the range: alginate/PVA > chitosan > alginate > alginate/chitosan (Figure 10), such as the temperature and pH stabilities. While the free lipase lost 60% of its activity after 10 days of storage, the immobilised lipase preserved more than 80% of its activity. It is noteworthy that the lipase encapsulated into chitosan-coated Ca-alginate beads conserved 89% of its activity after 30 days of storage, while the free lipase was almost completely inactivated. Nevertheless, a rapid drop in lipase activity was observed, using the hybrid Ca-alginate/PVA beads as enzyme supports. This fact could be attributed to enzyme leakage, due to PVA dissolution.

## Conclusions

The comparison of the performances of the four hydrophilic polymers – Ca-alginate/PVA, chitosan, Ca-alginate, and chitosan-coated Ca-alginate – demonstrated that the chitosan-coated Ca-alginate beads could be considered as a promising matrix for lipase immobilisation. Even if the loading efficiency of the chitosan-coated Ca-alginate beads was inferior to this of the Ca-alginate beads, they demonstrated better performance characteristics in terms of enzyme immobilisation yield, pH, thermal, and storage stability, and enzyme activity retention compared to the other selected polymers. The presented studies may be of potential value for the production of esters in the reactions of transesterification using immobilised lipases.
